# A Noddings’ caring theory-based intervention to enhance coping with death competence in advanced lung cancer patients: a randomized controlled trial

**DOI:** 10.1007/s00520-026-10739-2

**Published:** 2026-05-08

**Authors:** Jing Li, Runqin Huang, Xian Luo, YongJia Zhan, Chao Yan, Shenghuan Yang, Xiaojing Tian, Guoping He, Yonghong Li

**Affiliations:** 1 Nursing Department, the Second Affiliated Hospital of Zunyi Medical University, Zunyi City, Guizhou, China; 2 Nursing Department, the Affiliated Hospital of Zunyi Medical University, Zunyi City, Guizhou, China; 3 Nursing Department, Guizhou University of Traditional Chinese Medicine Second Affiliated Hospital, Guiyang, China; 4 Nursing Department, Guizhou Aerospace Hospital, Zunyi City, Guizhou, China

**Keywords:** Advanced lung cancer, Noddings’ caring theory, Coping with death competence, Death education

## Abstract

**Purpose:**

This randomized controlled trial aimed to evaluate the effects of a Noddings’ caring theory-based intervention on coping with death competence (CDC), death attitudes, and psychological distress in patients with advanced lung cancer.

**Methods:**

Seventy inpatients were randomly allocated to the intervention group (*n* = 35) or the control group (*n* = 35). The 3-week intervention comprised modeling, dialogue, practice, and recognition phases. Outcomes, including CDC, attitude toward death, anxiety, and depression, were assessed at baseline (T0), post-intervention (T1), and 1-month follow-up (T2).

**Results:**

No significant between-group differences were observed at baseline. Following the intervention, patients in the intervention group demonstrated significantly higher CDC total and subscale scores, more positive death attitudes, and lower anxiety and depression scores than those in the control group at both post-intervention and 1-month follow-up.

**Conclusion:**

The Noddings-based program effectively enhanced death-coping competence, improved death attitudes, and reduced psychological distress in patients with advanced lung cancer.

**Trial registration:**

ChiCTR2600118530, 2/6/2026 retrospectively registered.

**Supplementary Information:**

The online version contains supplementary material available at 10.1007/s00520-026-10739-2.

## Introduction

Global cancer statistics in 2022 show that the incidence rate (12.4%) and mortality rate (18.7%) of lung cancer are increasing year by year, and the incidence rate and mortality rate of lung cancer in China are at the top of the list of malignant tumors, with an overall survival rate of less than 20% at 5 years [1, 2]. Facing the fact that the disease is incurable and the survival rate is low, patients with advanced lung cancer are unable to face and cope with the impending death, resulting in sadness, despair, and other negative emotions, and some of them may even have aggressive behaviors [3]. Chinese culture often treats discussions of life and death as taboo, particularly during illness, which makes it difficult for patients to broach the topic with family members. So patients often fail to talk about life and death with their family members. It is difficult to open the topic of death, resulting in the quality of death being at a low to medium level [4]. The results of a meta-analysis indicated that the level of depression and anxiety in cancer patients was significantly associated with disease-specific and all-cause mortality [5]. In addition, one study reported that 40% of lung cancer patients experienced negative emotional states such as anxiety and low mood [6]. These findings suggest that emotional distress may be associated with confronting death and may warrant greater attention in the context of advanced lung cancer. In the present study, we use the Hospital Anxiety and Depression Scale, a well-validated screening tool for assessing the severity of anxiety and depressive symptoms in non-psychiatric hospital populations [7, 8]. Accordingly, when we refer to “anxiety” and “depression” in this study, we are referring to the self-reported emotional states—specifically, elevated levels of anxiety and low mood—related to the patient’s hospital experience and health condition.

The topic of coping with death has its roots in death education research that emerged in the twentieth century. In 1980, American scholar Bugen [9] conducted a death education program among college students to improve their ability to cope with their own deaths and the deaths of others and subsequently developed the 30-item Coping with Death Scale to enable participants to assess their coping abilities following death education training. Bugen conceptualized “coping with death” as a set of skills and abilities to face death-related situations. Subsequently, Robbins [10] further refined this construct as “death competency,” defining it as “a range of human skills and capabilities in dealing with death, as well as our beliefs and attitudes about these capabilities,” and emphasized its multidimensional nature and utility in evaluating death education outcomes. Currently, there is no universally accepted terminology to describe individuals’ capacity to deal with death-related challenges. In the present study, we adopt the term “coping with death competence” (CDC) to reflect both the process of coping and the underlying sense of competence when facing death-related situations [11]. It is also important to situate CDC within the broader theoretical framework of self-efficacy. Bandura’s social cognitive theory defines self-efficacy as an individual’s belief in their capacity to execute actions required to achieve specific outcomes [12]. Robbins [10] argued that death competency can be understood as perceived self-efficacy in coping with death-related situations. CDC thus extends the self-efficacy concept by integrating not only the belief in one’s capabilities but also the actual skills, attitudes, and emotional self-regulation that enable effective coping. The notion of death competency has been psychometrically operationalized as Bugen’s Coping with Death Scale [13]. It is important to distinguish this construct from the related but distinct concept of “death literacy.” Death literacy primarily refers to the knowledge and skills a person needs to access, understand, and make informed choices about end-of-life options [14]. In contrast, CDC goes beyond knowledge, encompassing the application of knowledge in practice as well as the recognition and management of one’s own emotional responses in the context of caring for others or facing one’s own death. Thus, a person may possess death literacy without necessarily being able to translate that knowledge into effective coping, particularly in emotionally charged situations. CDC therefore captures the integration of knowledge, self-awareness, and emotional regulation that enables meaningful action in the face of death.

Death education is educational content that focuses on introducing death-related knowledge and guiding the educated to correctly perceive death and cope with fatal events [15]. Studies have shown that death education can improve the way patients deal with death and is an effective means to improve cancer patients’ attitudes toward life and death and reduce their negative emotions [16]. While death education exists in China, its efficacy is limited by unidirectional knowledge transfer.

Noddings’ educational philosophy mentions that education should not only rely on the unilateral output of “indoctrination” and “filling in the blanks” but also on the establishment of a caring relationship between the educator and the educated, so as to make the educated feel the other party’s concern, and thus bring the educated into fully playing the educator’s ability to realize their own feelings. Noddings’ theory emphasizes caring relationships as foundational for transformative education [17]. Unlike didactic death education, this approach uses modeling, dialogue, practice, and recognition to foster authentic engagement with mortality. Several studies have demonstrated the applicability of Noddings’ caring theory in disease knowledge education [18] and improving patients’ quality of life [19]. The purpose of this study was to evaluate the effects of a CDC program based on Noddings’ caring theory on CDC, attitudes toward death, and negative emotions in patients with advanced lung cancer. To our knowledge, this is the first randomized controlled trial to apply the comprehensive framework of Noddings’ caring theory to a death-competence intervention for advanced lung cancer patients.

## Methods

### Study design and participants

This study was a prospective randomized controlled trial conducted in the Department of Thoracic Oncology at a tertiary general hospital in Guizhou Province, China. Randomization was performed using a computer-generated sequence by SPSS. The study was conducted in accordance with the Declaration of Helsinki after informing the subjects about the purpose of the intervention and the content of the study, and after the patients gave their informed consent and voluntarily signed an informed consent form. Ethical approval was obtained from the Biomedical Ethics Committee of the hospital (KLLY-2022-060). All patient information was anonymized and kept confidential.

**Fig. 1:**
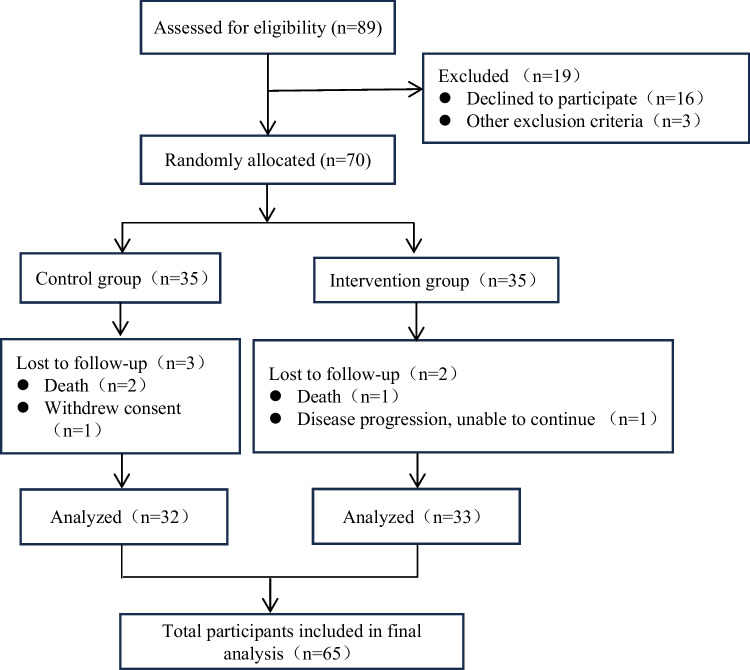
Flowchart for inclusion of study subjects

The study participants were patients with advanced lung cancer who were hospitalized in the Department of Thoracic Oncology from April 2023 to September 2023. Inclusion criteria are as follows: (1) stages III–IV lung cancer (TNM 8th ed.) [2], (2) age ≥ 18 years, (3) ability to complete the intervention independently, (4) awareness of diagnosis, (5) hospitalization ≥ 2 weeks, and (6) informed consent to voluntarily participate. Exclusion criteria are as follows: (1) patients with other serious diseases in combination, (2) patients with a pre-existing mental health disorder diagnosis, (3) language, hearing, and comprehension disorders, and (4) patients who were participating in a similar study. Disengagement criteria are as follows: (1) subject’s death or loss of visit during the study period, (2) subject’s inability to complete the study as scheduled during the study period, and (3) patients who withdrew from the study due to other force majeure factors. This study utilized G*Power (version 3.1.9.7) and, in reference to relevant research [20], set the parameters as follows: effect size = 0.25, *α* = 0.05, power = 0.80, number of groups = 2, number of repeated measures = 3, within-group intercorrelatio*n* = 0.5, and correction for the spherical hypothesis = 0.5. The total sample size was calculated to be *N* = 44. To account for a 25% dropout rate, the target sample size for this study was at least 55 participants. Ultimately, a total of 70 patients were enrolled. The flowchart is shown in Fig. 1.

### Interventions

The members of the research team were pre-trained, including one master’s degree nursing student who was responsible for implementing the intervention protocol and collecting and analyzing data; one mentor who was the team leader and supervised and guided the intervention process; one counselor who assisted the researcher in addressing the negative emotions of the patients; one oncologist who was responsible for explaining the disease knowledge to the patients and monitoring the disease status of the patients; and two oncology nurses who were responsible for patient health education during the study.

### Intervention group

The intervention protocol was developed based on a preliminary current situation survey and two rounds of expert consultations (See Appendix 1). The 3-week intervention program incorporated the four core elements of Noddings’ caring theory: modeling, guiding patients to reflect on their own mortality through structured questions and by providing exemplars of positive coping that help reconceptualize death from a source of fear to a natural part of life. Dialogue, facilitating structured discussions on the meaning of disease- and death-related anxiety, encouraging patients to express their fears and hopes, serving as an open-ended conversation grounded in Noddings’ emphasis on authentic communication. Practice, transforming abstract death concepts into concrete actions by using educational videos, picture books, and guided “reverse life education” with families, enabling patients to shift from passive recipients to active caregivers, thereby building practical skills and self-efficacy. Confirmation, employing self-reflection checklists for patients to review their progress in coping with death. The program consisted of six sessions, each lasting 40–60 min, conducted twice weekly. Educational materials were delivered in the form of videos, pamphlets, and images.

### Control group

The control group received standard nursing care for lung cancer patients in the Department of Thoracic Oncology. After enrollment, patients received standard care in the form of one-on-one conversations, including education on disease knowledge, diet, radiotherapy and chemotherapy, analgesic medication, and psychological care, twice a week for 15–20 min per session. The intervention lasted for 3 weeks.

#### Outcome measures

All patients completed a general information questionnaire at enrollment (See Appendix 2). The primary outcome was CDC, while secondary outcomes included attitudes toward death, as well as anxiety and depression. The questionnaires returned on the spot were verified in a timely manner to ensure that they were complete and logically correct. At the follow-up stage, the questionnaires were completed face-to-face for patients who returned to the hospital for review or treatment, and by telephone for those who did not return.

### Coping with death scale (CDS)

The CDS was selected for this study. While a shorter version [21] and other death-related competency scales [10, 22] have been validated in Chinese populations, these were either designed for healthcare professionals or reduced the construct to a unidimensional structure. Given our patient-based sample and our interest in multidimensional coping, the original 30-item version was deemed most appropriate. The Chinese version of the CDS was used for measurement, with permission obtained from the author(s). The scale consists of 28 items across six dimensions, rated on a 7-point Likert scale (1 = completely disagree, 7 = completely agree) [23]. Higher scores indicate stronger CDC. The scale demonstrated good reliability in this study (Cronbach’s *α* = 0.848; see Appendix 3).

### Death attitude profile-revised (DAP-R)

Attitudes toward death were assessed using the Chinese version of the DAP-R scale, which consists of 32 items covering five dimensions: fear of death, death avoidance, natural acceptance, approach acceptance, and escape acceptance [24]. Responses are rated on a 5-point Likert scale (1 = strongly disagree, 5 = strongly agree). The scale does not yield a total score; higher scores in a given dimension reflect a stronger tendency toward that particular attitude (Cronbach’s *α* = 0.875; see Appendix 4).

### Hospital anxiety and depression scale (HADS)

The patient's psychological state was screened using the HADS. The scale consists of 14 items, divided into two subscales: anxiety (7 items) and depression (7 items). A 4-point Likert scale (0–3) is used, and the sum of the items in each subscale indicates the level of anxiety and depression. In this study, a score of 10 was used as the cut-off value for anxiety and depression in patients with advanced lung cancer [25]. (See Appendix 5).

### Data analysis

Data analysis was performed using SPSS version 29.0. The study used descriptive statistics. The Shapiro–Wilk test indicated that the data were normally distributed. The Chi-square test was used for count data; two independent samples *t*-test were used for measures that conformed to a normal distribution with chi-square, and the Mann–Whitney *U* test was used for measures that were not normally distributed. Generalized estimating equations were used to analyze the data at three time points before and after the intervention; *p* < 0.05 was used as the level of significance.

## Results

### Characteristics of patients

A total of 70 patients participated in this study, and they were randomly assigned to the intervention group (*n* = 35) and the control group (*n* = 35). Attrition rates were 5.71% (intervention group) and 8.57% (control group), primarily due to death or clinical deterioration. Therefore, 65 patients were finally included for statistical analysis. In the control group, there were 28 (87.50%) males and four (12.50%) females, with a mean age of (56.63 ± 4.82) years; in the intervention group, there were 27 (81.80%) males and six (18.20%) females, with a mean age of 55.58 ± 4.60 years. No statistically significant difference was found between the baseline data of the two study groups (*P* > 0.05), and the demographic information of the patients is shown in Table 1.

**Table 1:** Comparison of the patients’ demographic characteristics between the intervention and control groups

Characteristic		Control group (*n* = 32)	Intervention group (*n* = 33)	Statistic	*P*
Gender	Male	28 (87.5)	27 (81.8)	0.085^b^	0.771
	Female	4 (12.5)	6 (18.2)		
Age (years)	< 60	22 (68.7)	27 (81.8)	1.495^a^	0.221
	≥ 60	10 (31.3)	6 (18.2)		
Ethnicity	Han Chinese	26 (81.2)	29 (87.9)	0.157^b^	0.692
	Minority	6 (18.8)	4 (12.1)		
Religious affiliation	Yes	3 (9.4)	1 (3.0)	0.300^b^	0.584
	No	29 (90.6)	32 (97.0)		
Literacy	Elementary school and below	9 (28.1)	10 (30.3)	− 0.122^c^	0.903
	Middle school	17 (53.2)	17 (51.5)		
	High school	5 (15.6)	4 (12.1)		
	University and above	1 (3.1)	2 (6.1)		
Place of residence	Town	14 (43.8)	16 (48.5)	0.147^a^	0.702
	Rural	18 (56.2)	17 (51.5)		
Smoking history	Yes	25 (78.1)	27 (81.8)	0.138^a^	0.710
	No	7 (21.9)	6 (18.2)		
Occupation	Laborer	6 (18.8)	5 (15.1)	-^d^	0.846
	Farmers	11 (34.3)	11 (33.3)		
	Civil servant	1 (3.1)	2 (6.1)		
	Retirement	4 (12.5)	2 (6.1)		
	Other	10 (31.3)	13 (39.4)		
Exposure to death of a close relative in the last 5 years	Yes	14 (43.8)	15 (45.5)	0.019^a^	0.890
	No	18 (56.2)	18 (54.5)		
Talked to others about the topic of death	Yes	5 (15.6)	9 (27.3)	1.304^a^	0.253
	No	27 (84.4)	24 (72.7)		
Primary caregiver	Spouse	19 (59.4)	16 (48.5)	-^d^	0.892
	Child	4 (12.5)	5 (15.1)		
	Other	2 (6.2)	2 (6.1)		
	Unaccompanied	7 (21.9)	10 (30.3)		
Monthly income	≤ 2000	20 (62.5)	22 (66.7)	− 0.508^c^	0.612
	2001–4000	9 (28.1)	10 (30.3)		
	≥ 4001	3 (9.4)	1 (3.0)		
Payment method	Resident medical insurance	27 (84.4)	29 (87.9)	0.002^b^	0.960
	Employee medical insurance	5 (15.6)	4 (12.1)		
Disease diagnosis duration	< 6 months	20 (62.5)	13 (39.4)	− 1.779^c^	0.075
	6 months - 1 year	5 (15.6)	8 (24.2)		
	＞ 1 year	7 (21.9)	12 (36.4)		
Clinical staging	Stage III	13 (40.6)	16 (48.5)	0.406^a^	0.524
	Phase IV	19 (59.4)	17 (51.5)		

^a^Pearson’s *X*^2^ test

^b^Continuous corrected *X*^2^ test

^c^Mann–Whitney *U* test

^d^Fisher’s exact test

### CDS

At baseline, no significant differences were observed between groups in total CDC scores or subscale scores (*P* > 0.05). The intervention group demonstrated significantly greater improvements in total CDC scores and all subscale scores over time compared to the control group, and the results are shown in Table 2.

**Table 2:** CDC scores (mean ± SD)

Dimension	Group	T0	T1	T2	Group * Time
Total CDC score	Control group (*n* = 32)	110.69 ± 15.39	113.75 ± 10.29	115.63 ± 9.78	< 0.001
	Intervention group (*n* = 33)	110.97 ± 15.74	127.33 ± 13.39	131.18 ± 13.41	
	*t*	− 0.073	− 4.577	− 5.330	
	*P*	0.942	< 0.001▲	< 0.001▲	
Dimension of ability to communicate with others about dying or death	Control group (*n* = 32)	32.28 ± 7.04	32.97 ± 4.94	33.47 ± 4.91	< 0.05
	Intervention group (*n* = 33)	32.45 ± 6.08	36.42 ± 5.57	37.67 ± 5.38	
	*t*	− 0.106	− 2.645	− 3.283	
	*P*	0.916	0.010▲	0.002▲	
Self-death acceptance dimension	Control group (*n* = 32)	15.88 ± 4.34	16.00 ± 3.56	16.31 ± 3.45	< 0.05
	Intervention group (*n* = 33)	15.67 ± 4.67	18.70 ± 3.55	19.15 ± 3.51	
	*t*	− 0.106	− 2.645	− 3.283	
	*P*	0.916	0.010▲	0.002▲	
Postmortem matters handling capacity dimension	Control group (*n* = 32)	15.59 ± 2.79	15.94 ± 2.37	15.97 ± 2.35	< 0.01
	Intervention group (*n* = 33)	15.24 ± 2.54	18.09 ± 3.14	18.48 ± 2.88	
	*t*	0.531	− 3.117	− 3.850	
	*P*	0.597	0.003▲	< 0.001▲	
CDC dimensions	Control group (*n* = 32)	20.50 ± 2.30	20.78 ± 2.72	21.00 (18.25, 23.00)	< 0.05
	Intervention group (*n* = 33)	20.73 ± 3.19	22.18 ± 2.16	22.00 (21.50, 24.50)	
	*t/Z*	− 0.328	− 2.295	− 2.510^#^	
	*P*	0.744	0.025▲	0.012▲	
Self-Death perception and expression dimensions of competence	Control group (*n* = 32)	16.00 (15.00, 19.75)	18.13 ± 2.85	18.53 ± 2.58	< 0.05
	Intervention group (*n* = 33)	17.61 ± 3.46	20.58 ± 3.29	20.97 ± 3.41	
	*t/Z*	− 0.805^#^	− 3.207	− 3.242	
	*P*	0.421	0.002▲	0.002▲	
Life inspections dimensions of competence	Control group (*n* = 32)	9.00 (6.00, 12.00)	10.50 (8.25, 11.75)	11.00 (9.00, 12.00)	< 0.05
	Intervention group (*n* = 33)	9.27 ± 2.82	11.00 (10.00, 13.00)	12.00 (11.00, 13.00)	
	*Z*	− 0.311^#^	− 2.375^#^	− 3.058^#^	
	*P*	0.756	0.018▲	0.002▲	

The number sign (#) denotes *Z*-value and the black triangle (▲) indicates group comparison, *P* < 0.05

### DAP-R

The results showed that there was no statistically significant difference between the two groups in the scores of the dimensions of death attitude before the intervention (*P* > 0.05), and the differences in the scores of the dimensions of death attitude between the two groups when comparing the T1 and T2 time points were statistically significant (*P* < 0.05). There was an interaction between time and subgroups (*P* < 0.05) in the scores of the dimensions of attitude toward death between the two groups of patients, as shown in Table 3.

**Table 3:** Intergroup comparison of DAP-R in the two groups

Item	Group	T0	T1	T2	Intergroup effect	Within-group effect	Interaction effect
Fear of death dimension	Control group (*n* = 32)	22.03 ± 4.02	20.97 ± 3.82	19.75 ± 2.81	9.112 (0.003)	134.802 (< 0.001)	62.785 (< 0.001)
	Intervention group (*n* = 33)	22.18 ± 3.65	16.39 ± 4.81	15.91 ± 4.64			
	*t*	− 0.158	4.238	4.053			
	*P*	0.875	< 0.001▲	< 0.001▲			
Death avoidance dimension	Control group (*n* = 32)	17.50 (14.25, 19.75)	15.66 ± 4.57	15.56 ± 4.49	8.235 (0.004)	231.176 (< 0.001)	142.796 (< 0.001)
	Intervention group (*n* = 33)	18.00 (15.00, 20.00)	12.00 (8.00, 17.50)	10.36 ± 2.98			
	*t/Z*	− 0.303^#^	− 2.526^#^	5.482			
	*P*	0.762	0.012▲	< 0.001▲			
Natural acceptance dimension	Control group (*n* = 32)	15.88 ± 4.27	17.19 ± 3.73	18.88 ± 2.85	14.552 (< 0.001)	81.586 (< 0.001)	22.285 (< 0.001)
	Intervention group (*n* = 33)	16.09 ± 5.31	21.76 ± 2.11	22.12 ± 1.67			
	*t*	− 0.180	− 6.059	− 5.581			
	*P*	0.858	< 0.001▲	< 0.001▲			
Convergence to acceptance dimension	Control group (*n* = 32)	28.00 (21.25, 29.00)	29.78 ± 3.93	30.41 ± 3.21	32.642 (< 0.001)	154.698 (< 0.001)	30.867 (< 0.001)
	Intervention group (*n* = 33)	30.00 (19.00, 31.00)	36.55 ± 4.01	38.21 ± 3.32			
	*t/Z*	− 0.810^#^	− 6.865	− 9.625			
	*P*	0.418	< 0.001▲	< 0.001▲			
Escape from acceptance dimension	Control group (*n* = 32)	15.41 ± 3.80	15.78 ± 4.13	16.00 (13.00, 20.00)	8.829 (0.003)	28.747 (< 0.001)	7.289 (0.026)
	Intervention group (*n* = 33)	16.03 ± 3.35	20.00 (16.00, 21.00)	20.00 (16.50, 21.00)			
	*t/Z*	− 0.703	− 3.065^#^	− 3.381^#^			
	*P*	0.485	0.002▲	0.001▲			

The number sign (#) denotes *Z*-value and the black triangle (▲) indicates group comparison, *P* < 0.05

### HADS

Anxiety decreased significantly in the intervention group at T2 (8.03 ± 1.51; *P* < 0.001). Similar reductions occurred in depression at T2 (6.76 ± 2.33; *P* < 0.001). There was an interaction effect between time and subgroups (*P* < 0.001) for the differences in HADS total scores and anxiety and depression scores between the two groups, as shown in Table 4.

**Table 4:** Intergroup comparison of HADS in the two groups

Item	Group	T0	T1	T2	Intergroup effect	Time effect	Interaction effect
Anxiety	Control group (*n* = 32)	11.00 (8.00, 12.75)	10.22 ± 2.25	10.66 ± 2.44	7.548 (0.006)	43.715 (< 0.001)	45.240 (< 0.001)
	Intervention group (*n* = 33)	11.00 (8.00, 13.00)	8.61 ± 1.52	8.03 ± 1.51			
	*t/Z*	−0.325^#^	3.372	5.192			
	*P*	0.745	0.001▲	< 0.001▲			
Depression	Control group (*n* = 32)	11.44 ± 2.58	10.78 ± 2.85	10.59 ± 2.78	14.795 (< 0.001)	128.916 (< 0.001)	65.549 (< 0.001)
	Intervention group (*n* = 33)	11.85 ± 2.51	7.45 ± 2.59	6.76 ± 2.33			
	*t*	−0.651	4.932	6.033			
	*P*	0.518	< 0.001▲	< 0.001▲			
Total HADS score	Control group (*n* = 32)	22.13 ± 4.06	21.00 ± 3.77	21.25 ± 3.76			
	Intervention group (*n* = 33)	22.82 ± 3.67	16.06 ± 3.61	14.79 ± 3.21	17.810 (< 0.001)	202.856 (< 0.001)	129.679 (< 0.001)
	*t*	−0.722	5.395	7.462			
	*P*	0.473	< 0.001▲	< 0.001▲			

The number sign (#) denotes *Z*-value and the black triangle (▲) indicates group comparison, *P* < 0.05

## Discussion

This study is the first randomized controlled trial to evaluate the effects of a Noddings’ caring theory-based intervention on CDC in patients with advanced lung cancer. The results demonstrate that a structured, relationally centered program can significantly enhance CDC, improve attitudes toward death, and reduce psychological distress. These findings contribute to the growing body of evidence supporting the value of death education in oncology care.

### Significant and sustained improvement in CDC

Our study demonstrated that the Noddings-based intervention led to a significant and sustained improvement in CDC scores among patients with advanced lung cancer. This finding aligns with prior research showing that death education can enhance coping abilities [26, 27] and extends it by revealing a critical mechanism: the sequential, relational process of modeling, dialogue, practice, and confirmation may be particularly effective in fostering this competence.

While previous studies have highlighted the importance of life meaning [28] or experiential learning [29] in improving death attitudes, our results suggest that the relational context is a crucial catalyst. This active role-taking, enabled by a trusting relationship with the facilitator, is a unique contribution of the Noddings framework that is often absent in didactic death education programs. However, our sample was predominantly male (approximately 85%). This gender imbalance limits our ability to examine whether the intervention’s effects differ by gender. Some studies suggest that females may exhibit higher levels of death anxiety but also greater emotional expressiveness, which could influence engagement with dialogue-based intervention components [26]. Conversely, other research has found no significant gender differences in death competence [28]. Future studies with more balanced gender representation are needed to explore potential gender-specific effects. During the “confirmation” component, the researcher used a self-reflection checklist to guide patients in reviewing their progress in coping with death and provided timely recognition. As Han et al. [30] noted in their study on peer support, when individuals’ positive changes are acknowledged by others, such recognition reinforces adaptive behaviors and enhances self-efficacy. By recognizing and affirming their own growth, patients likely internalized a new, more meaningful self-narrative, which in turn bolstered their perceived competence to cope with death.

Furthermore, the improved ability to communicate about death, as evidenced by the CDC subscale scores, suggests that our intervention could serve as a precursor to, or a component of, advance care planning (ACP) initiatives. Fan et al. [31] demonstrated that ACP enables individuals to cope better with death by articulating their wishes. Our program may lower the initial emotional and cultural barriers to such conversations, potentially increasing the uptake and quality of ACP.

### Positive shift in death attitudes

This study showed that the intervention group had significantly higher scores in the dimensions of “natural acceptance” and “approach acceptance” compared to the control group, while scores in the dimensions of “fear of death” and “death avoidance” were significantly lower. These findings indicate that the intervention not only helped patients reduce negative emotions toward death, but more importantly, facilitated their acceptance of death.

Unlike previous studies, the intervention in this study was designed based on Noddings’ caring theory, offering a new perspective for understanding the mechanisms underlying changes in death attitudes. We found that the intervention strategies of modeling and dialogue played a key role in facilitating attitude change. In the “modeling” component, stories of patients who had coped positively with death provided other patients with relatable learning experiences. According to Bandura’s self-efficacy theory [12], observing others successfully cope with challenges can enhance the observer’s own belief in their ability to do the same. The magnitude of improvement in the “approach acceptance” dimension observed in our intervention group was larger than that reported in previous studies conducted in Chinese populations [26]. This discrepancy may be partly attributable to differences in study design, including the single-center design of our trial, which may have introduced expectancy effects; the higher intensity of our intervention (six 40- to 60-min sessions) compared with some previous studies. Additionally, the lower baseline levels in our advanced cancer cohort may have created greater room for improvement. Moreover, in the context of Chinese culture, traditional religious beliefs are not prevalent, yet approach acceptance scores still increased. This suggests that approach acceptance may stem not only from religious beliefs but also from a reaffirmation of life’s meaning and a recognition of one’s legacy within the family. Shaw et al. [32] noted when patients have the opportunity to express their feelings about death within a safe, caring relationship, death shifts from being an “unspeakable taboo” to a “discussable topic.”

### Significant alleviation of psychological distress

If not addressed promptly, persistent anxiety and depression can compromise immunity and lower quality of survival [33]. This study demonstrated that the intervention based on Noddings’ caring theory significantly reduced anxiety and depression levels in patients with advanced lung cancer. Notably, at T2, the intervention group’s anxiety score (8.03 ± 1.51) and depression score (6.76 ± 2.33) fell below the threshold typically considered “clinically significant” on the HADS scale. The findings of this study are consistent with those of Zhang et al. [26].

The intervention in this study, grounded in Noddings’ caring theory, provides a new explanatory framework for understanding the mechanisms underlying the alleviation of psychological distress. We propose that the “dialogue” and “confirmation” components played key roles. As Ye [34] noted in the application of Watson’s caring theory, when patients’ psychological needs are listened to and validated, feelings of loneliness and helplessness are significantly reduced. In this study, many patients expressed after the intervention that “finally someone is willing to listen to me,” suggesting that the experience of being heard is itself a powerful form of psychological support. Research by Rezaei et al. [35] also showed that allowing patients to express end-of-life concerns during initial treatment conversations can effectively reduce psychological burden. Furthermore, the effects of the intervention were sustained at the 1-month follow-up, suggesting that the “reverse life education” that patients engaged in with their families may have played an ongoing role in maintaining these improvements.

The findings have immediate implications for clinical practice. Research suggests that psychological interventions remain effective even in the terminal stages of illness. This study, together with previous research [16], indicates that structured, caring-based dialogue does not exacerbate distress; rather, it alleviates suffering by normalizing and verbalizing fear. A shift from merely providing information to facilitating short, guided conversations about end-of-life fears and hopes could be a powerful, low-intensity intervention to reduce patient distress. Furthermore, empowering patients to share their wishes or life lessons with family members may not only enhance their own CDC but also strengthen family bonds and reduce the burden on caregivers during the bereavement phase.

## Limitations

Several limitations should be acknowledged when interpreting the findings of this study. First, this study was conducted at a single center, which may limit the generalizability of the findings. Second, the CDS, although validated in Chinese populations, is a self-report instrument and may be subject to social desirability bias; in addition, our analysis did not account for several variables that may influence CDC and responsiveness to the intervention. Disease-related factors such as symptom burden, functional status, and prior experience with death education may also have contributed to baseline differences in CDC, although randomization minimized systematic bias. Future studies should consider using qualitative methods to complement self-reported data and collecting these additional variables to allow for more detailed subgroup analyses. Third, baseline analysis showed no significant association between age and CDC scores (*P* > 0.05). Nevertheless, given prior evidence of age-related differences in death competence, future studies with larger samples should explore whether age moderates intervention effects using continuous age analyses or finer age categorizations.

## Conclusions

The four steps of modeling, dialogue, practice, and recognition can lead patients to engage with the topic of death, think about death, recognize and cope with death. This approach significantly enhances death coping competence and psychological well‑being in patients with advanced lung cancer, providing an operational, replicable, and theory‑grounded framework of relational care for death education in oncology nursing, thereby opening a new pathway for its integration into practice.

## Supplementary Information

Below is the link to the electronic supplementary material.

ESM1 Appendix 1 (DOCX 22.9 KB)

ESM2 Appendix 2 (DOCX 19.9 KB)

ESM3 Appendix 3 (DOCX 24.2 KB)

ESM4 Appendix 4 (DOCX 22.9 KB)

ESM5 Appendix 5 (DOCX 19.6 KB)

## Data Availability

No datasets were generated or analyzed during the current study.
