# Supplementary material for: A Noddings’ caring theory-based intervention to enhance coping with death competence in advanced lung cancer patients: a randomized controlled trial
Source: Support Care Cancer. 2026 May 8;34(6):518. doi: 10.1007/s00520-026-10739-2 (PMC13156160; doi:10.1007/s00520-026-10739-2)
Supplement: Supplementary file 1 — Appendix 1 (DOCX 22.9 KB) [file 520_2026_10739_MOESM1_ESM.docx]

| Stage | Intervention Approach | Intervention content |
| --- | --- | --- |
| Example | Day 1-3 of the 1st week of enrollment  ①Intervention personnel: researcher, responsible nurse  ②Intervention time: about 30-60mins  ③Place of intervention: conference room  ④ Intervention form: group intervention (5-10 people) | 1. Play the video: Lee Kai-Fu's "Life to Death" documentary about Lee's journey during treatment and his thoughts on life in the face of death. |
|  |  | 2. Introduce advanced lung cancer patients with positive mindset in the department and encourage patients to learn the power of role models. |
|  |  | 3. Present lectures: patients with advanced lung cancer share their own experience of treatment, psychological feelings and adjustment of mindset, so as to alleviate their negative emotions and enhance patient care. |
|  |  | 4. Medical staff will answer the questions raised by the patients together with the "role model" patients to show the care of doctors and patients.  5. Excerpts from recommended books: Lee Kai-Fu's "Living Toward Death, My Death Credits": "If there are only 100 days left in life", "Life is the toughest tutor"; Ling Zhijun's "Handbook of Rebirth": "Listen to your own body", "Listen to your own body", "Listen to your own body", "Listen to your own body", "Listen to your own body", "Listen to your own body", "Listen to your own body". Body", "Scenery on the Road", and "Healing in the Non-Medical Sense". |
| Dialogue | Enrollment Week 1 Days 4-7  ①Interventionist: researcher  ②Intervention time: about 30min  ③Place of intervention: ward/meeting room  ④Intervention format: face-to-face | 1. What was your psychological process after the disease? How do you cope with negative emotions? |
|  |  | 2. What concerns, expectations and blessings do you have for your family after your illness? |
|  |  | 3. Do you need your family, friends or others to do anything for you? |
|  |  | 4. How do you feel about the end of life? |
|  |  | 5. If you were facing imminent death, how would you plan for the rest of your life? |
|  |  | 6. What experiences of illness would you like to share with others? |
| Practice | Day 1-3 of the 2nd week of enrollment  ①Intervention personnel: researcher, responsible nurse  ②Intervention time: about 60min  ③Place of intervention: ward  ④Intervention format: one-on-one | 1. Play the animated video "All the people you've ever met will experience a death" (Beep App). |
|  |  | 2. Play Professor Wang Yunling's public lecture "Death culture and life and death education" - the meaning of death, fear and anxiety of death, funeral etiquette (China University MOOC APP). |
|  |  | 3. Patients and their family members will learn the picture book "I Love You Forever" together, encouraging patients and their family members to express their love for each other, and promoting the opening of communication on the topic of end-of-life. |
|  | 4-7 days in the 2nd week of enrollment  ①Intervention personnel: researcher, responsible nurse  ②Intervention time: about 60min  ③Location of intervention: ward  ④ Intervention form: one-on-one | 4. The Four Ways of Life: Through the video excerpt of "The Unbearable Weight of Life - Life and Death from the Medical Perspective", patients are guided to apologize to their family members→ to say thank you→ to say love→ to say goodbye, and to care for their family members in the reverse direction. |
|  |  | ① Behavioral care: choose the rhythm of gentle music, guide the patient to hold the family's hand, embrace the family, listen to the family's true inner thoughts, improve mutual understanding. |
|  |  | ② Love Forever: according to the patient's preference, diversified ways to collect what the patient wants to say to his family (love, blessings, wishes, expectations), such as audio, video, cards, etc., as a souvenir for the family. |
|  |  | ③Spiritual comfort: Through their own experience of the disease (feelings of illness, coping, needs), patients inspire their family members to value life, and then help them to establish a correct understanding of the face of death. |
| Recognition | 3rd week of enrollment  ①Intervention personnel: the researcher  ② Intervention time: about 20min  ③Intervention format: one-on-one by phone | 1. "Self-recognition questionnaire", patients answer "yes" and "no": |
|  |  | ①Do you recognize that life and death are natural laws? |
|  |  | ②Do you recognize that life and death are interdependent? |
|  |  | ③Do you think that thinking about death will cause you distress? |
|  |  | ④ Do you think that thinking about death causes you distress? ④ Can you honestly express your love to your family? |
|  |  | ⑤ Do you take the initiative to communicate with your family about your afterlife? |
|  |  | ⑥Can you accept death? |
