# Supplementary material for: A Noddings’ caring theory-based intervention to enhance coping with death competence in advanced lung cancer patients: a randomized controlled trial
Source: Support Care Cancer. 2026 May 8;34(6):518. doi: 10.1007/s00520-026-10739-2 (PMC13156160; doi:10.1007/s00520-026-10739-2)
Supplement: Supplementary file 2 — Appendix 2 (DOCX 19.9 KB) [file 520_2026_10739_MOESM2_ESM.docx]

Dear Patient:

Greetings! Thank you for taking the time to complete the questionnaire! This questionnaire will investigate your general situation and will not disclose your personal information. Please answer the survey questions truthfully according to your actual situation, and hit "√ " in the content that meets your situation, thank you for your cooperation!

1、Age: ___.

2、Gender：□Male □Female

3、Nationality：□Han nationality □other ___.

4、Religion：□Yes □No

5、Educational level: □ elementary school and below □ junior high school □ high school □ university and above

6、Marital status: □unmarried □married □divorced □widowed

7、Children：□Yes□No

8、Location：□urban □rural

9、Smoking History: □Yes □No

10、Occupation：□Worker □Farmer□ Teacher□ Civil servant □Other _______.

11、Contact with death of close relatives in the past 5 years: □Yes □No

12、Talked about death with others：□Yes □No

13、Primary caregiver: □spouse □children □others □no companion

14、Monthly Income：□≤ $2000 □ 2001-4000 □≥ $4001

15、Payment method: □ Self-payment □ Resident's health insurance □ Employee's health insurance

16、Duration of disease diagnosis: ______.

17、Clinical Stage：□Stage III □ Stage IV
