# Supplementary material for: A Noddings’ caring theory-based intervention to enhance coping with death competence in advanced lung cancer patients: a randomized controlled trial
Source: Support Care Cancer. 2026 May 8;34(6):518. doi: 10.1007/s00520-026-10739-2 (PMC13156160; doi:10.1007/s00520-026-10739-2)
Supplement: Supplementary file 3 — Appendix 3 (DOCX 24.2 KB) [file 520_2026_10739_MOESM3_ESM.docx]

**Coping with death scale (CDS)**

Guideline: Death coping skills are a set of attitudes or skills that individuals use to deal with the death of themselves or others. The following entries are narratives related to death coping skills, where 1 score= completely disagree, 2 scores= disagree, 3 scores= relatively disagree, 4 scores= neutral, 5 scores= relatively agree, 6 scores= agree, and 7 scores= completely agree. Please tick the corresponding "□" according to your actual situation, thank you for your cooperation!

| Dimension | Entry | 1 | 2 | 3 | 4 | 5 | 6 | 7 |
| --- | --- | --- | --- | --- | --- | --- | --- | --- |
| Communicating with others end-of-life or death I know how to listen to others, including the terminally ill. | 1. I know how to listen to others, including listening to the terminally ill |  |  |  |  |  |  |  |
|  | 2. I know how to talk to children about death. |  |  |  |  |  |  |  |
|  | 3. I can spend time with the terminally ill if needed. |  |  |  |  |  |  |  |
|  | 4. I can help people with their thoughts and feelings about death and dying. |  |  |  |  |  |  |  |
|  | 5. I can talk to a friend or family member about his/her death. |  |  |  |  |  |  |  |
|  | 6. I am able to reduce the anxiety of those around me when they talk about death or dying |  |  |  |  |  |  |  |
|  | 7. I am able to communicate with the dying person. |  |  |  |  |  |  |  |
|  | 8. I am able to tell them how much I love them before I or someone else dies |  |  |  |  |  |  |  |
| Self-death acceptance | 9. Knowing that the fact that I am going to die will not have any effect on my life or behavior. |  |  |  |  |  |  |  |
|  | 10. I am prepared for death |  |  |  |  |  |  |  |
|  | 11. I am prepared for the dying process. |  |  |  |  |  |  |  |
|  | 12. I have recently realized that there is nothing (scary) about thinking about death. |  |  |  |  |  |  |  |
|  | 13. I can talk about my death with my family and friends. |  |  |  |  |  |  |  |
| What happens after death Ability to cope | 14. I am aware of the range of services offered by funeral directors. |  |  |  |  |  |  |  |
|  | 15. I am aware of the many options for disposing of a body. |  |  |  |  |  |  |  |
|  | 16. I am aware of all the manifestations of human grief. |  |  |  |  |  |  |  |
|  | 17. I am familiar with the preparations for a funeral service. |  |  |  |  |  |  |  |
| Death coping skills | 18. Death can be handled safely |  |  |  |  |  |  |  |
|  | 19. I know who to contact when a death occurs |  |  |  |  |  |  |  |
|  | 20. I will be able to cope with future losses (e.g., people, objects, etc.) |  |  |  |  |  |  |  |
|  | 21. I feel able to handle the death of someone close to me |  |  |  |  |  |  |  |
| Self-perception of death and ability to express it | 22. I have a positive view of death and the dying process |  |  |  |  |  |  |  |
|  | 23. I know where my fear of death lies |  |  |  |  |  |  |  |
|  | 24. I have recently changed my attitude toward life. |  |  |  |  |  |  |  |
|  | 25. I can express my fear of death |  |  |  |  |  |  |  |
|  | 26. I can clearly anticipate the coming of death and dying. |  |  |  |  |  |  |  |
| I have the ability to perceive life | 27. I am making the most of my present life. |  |  |  |  |  |  |  |
|  | 28. The quality of my life is more important than the length of my life |  |  |  |  |  |  |  |
