# Supplementary material for: A Noddings’ caring theory-based intervention to enhance coping with death competence in advanced lung cancer patients: a randomized controlled trial
Source: Support Care Cancer. 2026 May 8;34(6):518. doi: 10.1007/s00520-026-10739-2 (PMC13156160; doi:10.1007/s00520-026-10739-2)
Supplement: Supplementary file 4 — Appendix 4 (DOCX 22.9 KB) [file 520_2026_10739_MOESM4_ESM.docx]

**Death attitude profile-revised (DAP-R)**

Instructions for completion: Please read each item in the form and tick the box that best suits your situation. Thank you for your cooperation!

| Entry | 5 | 4 | 3 | 2 | 1 |
| --- | --- | --- | --- | --- | --- |
| 1. Death is a frightening experience |  |  |  |  |  |
| 2. I get anxious when I think about my own death |  |  |  |  |  |
| 3. I try not to think about death. |  |  |  |  |  |
| 4. I believe I will go to heaven when I die. |  |  |  |  |  |
| 5. Death will put an end to all my worries. |  |  |  |  |  |
| 6. Death is natural, undeniable and inevitable. |  |  |  |  |  |
| 7. The certainty that people will die bothers me. |  |  |  |  |  |
| 8. Death is the gateway to the land of bliss. |  |  |  |  |  |
| 9. Death allows me to escape from this horrible world. |  |  |  |  |  |
| 10. Whenever the thought of death enters my mind, I try to drive it away. |  |  |  |  |  |
| 11. Death is a relief from sorrow and suffering. |  |  |  |  |  |
| 12. I always try not to think of death. |  |  |  |  |  |
| 13. I believe that heaven is a better place than this world. |  |  |  |  |  |
| 14. Death is a natural part of the process of life. |  |  |  |  |  |
| 15. Death is a union with God (God, Buddha, etc.) and eternal bliss. |  |  |  |  |  |
| 16. Death is sure to bring about a new and glorious life. |  |  |  |  |  |
| 17. I neither fear nor welcome death. |  |  |  |  |  |
| 18. I have a strong fear of death. |  |  |  |  |  |
| 19. I avoid the thought of death altogether |  |  |  |  |  |
| 20. The question of whether there is life after death bothers me a great deal. |  |  |  |  |  |
| 21. The fact that death means the end of everything scares me. |  |  |  |  |  |
| 22. I hope to be reunited with my loved ones after death. |  |  |  |  |  |
| 23. I see death as a relief from the pain of this world. |  |  |  |  |  |
| 24. Death is just a part of the process of life |  |  |  |  |  |
| 25. I see death as a gateway to a place of eternal happiness. |  |  |  |  |  |
| 26. I try to avoid things related to death. |  |  |  |  |  |
| 27. Death provides a wonderful relief for the soul. |  |  |  |  |  |
| 28. When faced with death, I find comfort in the belief that there is life after death. |  |  |  |  |  |
| 29. I see death as a relief from the burdens of this life. |  |  |  |  |  |
| 30. Death is neither good nor bad. |  |  |  |  |  |
| 31. I look forward to life after death. |  |  |  |  |  |
| 32. The uncertainty of what will happen after death worries me. |  |  |  |  |  |
