# Supplementary material for: A Noddings’ caring theory-based intervention to enhance coping with death competence in advanced lung cancer patients: a randomized controlled trial
Source: Support Care Cancer. 2026 May 8;34(6):518. doi: 10.1007/s00520-026-10739-2 (PMC13156160; doi:10.1007/s00520-026-10739-2)
Supplement: Supplementary file 5 — Appendix 5 (DOCX 19.6 KB) [file 520_2026_10739_MOESM5_ESM.docx]

**Hospital Anxiety and Depression Scale(HADS)**

Instructions for completion: Please read each entry below and tick the box that best matches your status in the past month.

| Entry | Answer | Rating |
| --- | --- | --- |
| 1. I feel nervous (or distressed) | Not at all | 0 |
|  | Sometimes | 1 |
|  | Most of the time | 2 |
|  | Almost all the time | 3 |
| 2. I am still interested in things that used to interest me | Definitely the same | 0 |
|  | Not as much as before | 1 |
|  | Only a little | 2 |
|  | Basically nothing | 3 |
| 3. I feel a little bit scared, as if I have a premonition that something terrible is going to happen | Not at all | 0 |
|  | A little bit, but it doesn't distress me | 1 |
|  | Yes, but not too much | 2 |
|  | Very definitely and very seriously | 3 |
| 4. I am still able to laugh and see the good in things | I do this a lot | 0 |
|  | Not so much anymore | 1 |
|  | Definitely not too much now | 2 |
|  | Not at all. | 3 |
| 5. My heart is full of trouble | Occasionally | 0 |
|  | Sometimes, but not often | 1 |
|  | Often | 2 |
|  | Most of the time | 3 |
| 6. I feel happy | Most of the time | 0 |
|  | Sometimes | 1 |
|  | Not very often | 2 |
|  | Not at all | 3 |
| 7. I was able to sit comfortably and easily | Definitely | 0 |
|  | Often | 1 |
|  | Not very often | 2 |
|  | Not at all | 3 |
| 8. I don't care about my appearance anymore | I still care as much as I used to | 0 |
|  | I probably don't care very much | 1 |
|  | Don't care as much as I should | 2 |
|  | Definitely | 3 |
| 9. I fidgeted a bit, as if I felt compelled to be active | Not at all | 0 |
|  | Not a lot | 1 |
|  | Quite a lot | 2 |
|  | Very much indeed | 3 |
| 10. I'm optimistic about moving forward with everything | Pretty much do | 0 |
|  | Not exactly | 1 |
|  | Rarely | 2 |
|  | Almost never | 3 |
| 11. I suddenly realized that the sense of panic | Not at all | 0 |
|  | Not often | 1 |
|  | often | 2 |
|  | Very often indeed | 3 |
| 12. I seem to feel a gradual decline in mood | Not at all | 0 |
|  | Sometimes | 1 |
|  | Very often | 2 |
|  | Almost all the time | 3 |
| 13. I feel a little scared, as if one of my internal organs has gone bad | Not at all | 0 |
|  | Sometimes | 1 |
|  | Very often | 2 |
|  | Very often | 3 |
| 14. I can enjoy a good book or a good radio or TV program | Very often | 0 |
|  | Sometimes | 1 |
|  | Not always | 2 |
|  | Rarely | 3 |
